# Optimizing NP and PA roles to improve care access in an academic medical center: An organizational case study

**DOI:** 10.1097/01.JAA.0000000000000233

**Published:** 2025-07-29

**Authors:** Chris Ferron, Wendy Franklin, Hope Sellars, James Shamiyeh, Sandy Leake, Lacey Buckler, Jaime Lough, Amy Noecker, Zachary Hartsell

**Affiliations:** At the University of Tennessee Medical Center in Knoxville, TN, **Chris Ferron** is director of advanced practice, **Wendy Franklin** is an advanced practice nurse, **Hope Sellars** is an NP, **James Shamiyeh** is executive vice president and chief operating officer, and **Sandy Leake** is senior vice president and chief nursing officer. At SullivanCotter, Inc., in Chicago, IL, **Lacey Buckler** is consulting principal, **Jaime Lough** is managing consultant, **Amy Noecker** is managing principal, and **Zachary Hartsell** is managing principal. Zachary Hartsell is a member of the *JAAPA* editorial board and a *JAAPA* clinical editor. The authors have disclosed no other potential conflicts of interest, financial or otherwise, although SullivanCotter was engaged in a consulting relationship with the University of Tennessee Medical Center for the duration of this project.

**Keywords:** advanced practice providers, care access, care models, healthcare costs, healthcare service delivery, health systems science

## Abstract

The healthcare industry is in the midst of unprecedented change as hospitals and health systems nationwide balance the need to increase patient access to services with managing costs. Nurse practitioners and physician associates are well-positioned, and often overlooked, members of the healthcare team who can serve as resources for organizations to improve care access. Using an intentional and data-driven process, one academic medical center both improved patient access and increased revenue through a holistic care model redesign in three specialty areas, as described in this organizational case report.

As the healthcare system recovers from the pandemic and its lingering effects, patient access needs continue to grow. A recent Harris Poll of US adults (ages 18 years and older) found that only 10% of those surveyed gave the US healthcare system an “A” grade (on an “A” through “F” scale), whereas a majority (60%) gave it an average grade or worse (“C” to “F”).^1^ Of the reasons listed for how the system is not meeting people's needs, 31% of respondents reported that it takes too long to get an appointment, 13% said that finding the care they need is confusing and hard to navigate, and 13% stated that too few providers are available in the community to deliver the needed care. More than half (56%) of the people surveyed reported waiting 1 week or more for an appointment, with the average wait time found to be 3.9 weeks.^1^

These existing access issues, coupled with persistent physician workforce projections showing a shortage of between 13,500 and 86,000 physicians by 2036, create even greater constraints on patient access, both now and in the future.^2^ Organizations are now more than ever relying on advanced practice providers (APPs), including nurse practitioners (NPs) and physician associates (PAs), to fill the gap. Despite the record pace at which organizations are hiring NPs and PAs—and despite lessons learned from the pandemic regarding their deployment—the NP and PA role is often underutilized.^3^,^4^ According to SullivanCotter's 2023 Advanced Practice Provider Compensation and Productivity Survey—representing nearly 25% of the nation's APP workforce with data on nearly 125,000 individual providers—59% of organizations anticipated growing their NP and PA workforce within 12 months, with a median projected increase of 9.5%.^4^ The previous year's survey reported that nearly 86% of organizations use the same compensation practices for NPs and PAs in the same specialty.^5^ When comparing NP and PA productivity metrics in these surveys, only minimal differences were noted by specialty and overall: more specifically, PAs had approximately 1.3% higher work relative value units (wRVUs) and approximately 6% more encounters at median compared with NPs.^4^,^5^ This is likely due to differences in specialty distribution as opposed to differences in the roles.

Optimizing the role of NPs and PAs while continuing to support physicians is feasible and can result in a positive financial impact and increased patient access.^3^,^6^ A cross-sectional time-series study found that from 2013 to 2019, the increase in the number of NP and PA encounters was disproportionate to workforce growth and that, by 2019, NPs and PAs made up more than 25% of all billed encounters to Medicare.^7^ This increase in visits represented NP and PA expansion of care alongside physicians rather than replacement of physicians.^7^

Few organizations have approached care team optimization through the lens of the role of the NP and PA as a mechanism to expand access. This case study describes one academic medical center's journey to assess the current utilization of its NP and PA workforce and introduce an intentional process to engage the care team maximally.

## ORGANIZATION BACKGROUND

The organization described in this article is an academic medical center in the Southeast United States that had seen double-digit annual growth in the number of employed APPs during the past decade. Similar to other academic medical centers experiencing rapid expansion, processes and structures related to NPs and PAs at this organization were inconsistent. Among other issues, leadership recognized the need to optimize patient care delivery through more effective utilization of NPs and PAs. Between 2022 and 2023, the organization analyzed its workforce and found several areas for improvement: specifically, gaps existed in leadership and governance structure, productivity and performance expectations, and staff members' and physicians' recognition of the NP and PA role.

As part of its workforce analysis, the organization undertook a survey that found that 55% of APPs had considered leaving the organization within the previous 12 months.^8^ The two main reasons APPs considered leaving, in alignment with national results, included “minimal to no utilization as an APP” (1.69 OR) and “physicians do not understand APP roles or capabilities” (1.60 OR).^8^ Other factors identified through focus groups included a lack of professional respect and poorly defined roles. In addition, 15% of NPs and PAs noted that they were performing tasks that could be performed by others, which limited their role as providers.^9^ To better understand the opportunity for change within specific specialties, NPs and PAs were also asked about their utilization on a 5-point scale (*maximum, significant, moderate, minimal*, and *no utilization*). PAs and NPs in three specialties in particular reported feeling moderately utilized (or worse) as APPs within the academic medical center: cardiology (63%), oncology (63%), and hospital medicine (57%).^8^

**FIGURE 1.**
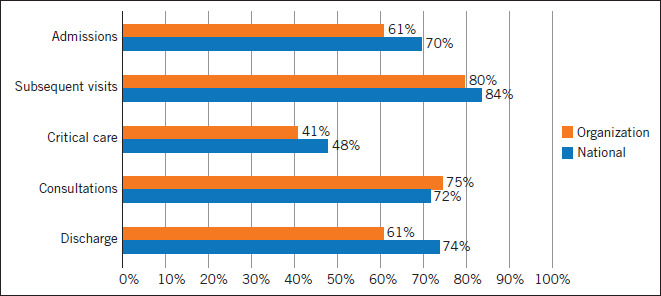
Proportion of inpatient services independently performed by APPs without simultaneous physician performance: Organizational data versus national benchmarks

Additional findings showed that, when compared with APPs at other academic medical centers, NPs and PAs within this organization were seeing fewer patients independently. For example, 61% of surveyed NPs and PAs reported discharging a patient without a physician present as compared with 74% nationally (Figure 1).^5^ Although the aggregate productivity of the NP and PA workforce as measured by wRVUs was in the 51st percentile, 10 specialties were below the market median, and five specialties were below the 25th percentile compared with national data.^4^

Increasing NP and PA productivity to meet the national median would represent approximately $1.1 to $3.6 million in additional collections for the health system annually (based on comparison with national productivity and utilizing the organization's historical rate of collections for NPs and PAs). With nearly 360 employed NPs and PAs, this organization's APP workforce also represented a significant payroll expense, yet the academic medical center lacked an intentional strategy for the deployment of its NPs and PAs.

## METHODS

This initiative aimed to enhance access through PAs and NPs within clinical departments with the greatest opportunity for improvement and an appropriate level of readiness for change. For this initiative, the organization partnered with a consulting firm, which provided data analysis and project management support. A steering committee was formed to identify the clinical departments with the greatest opportunity for enhancement. The steering committee was composed of the chief medical officer, chief nursing officer, top APP leader, and key leadership advisors from relevant service lines. Anecdotally, the consulting firm has found that the departments that have achieved the most success in this type of project are those that demonstrate both a cultural readiness for change and a significant quantifiable opportunity to increase revenue and/or access. Based on the survey findings and the consulting firm's experience, the departments selected for the first phases of the redesign work included cardiology, oncology, and hospital medicine, which had an appropriate level of perceived readiness for change based on interviews as well as potential opportunity to improve access. The project team, which reported to the steering committee, was formed and was led by the director of advanced practice and the consulting firm. The steering committee met monthly throughout the project and approved the final specialty implementation plans and proposed metrics.

Each specialty formed workgroups comprised of physicians, NPs, PAs, registered nurses (RNs), administrators, and medical assistants (MAs) who met over a series of four to five meetings. In addition to lending their experience regarding specialty-specific care models, the workgroups used a data-driven approach to:

- Review the current state of the care teams
- Define the ideal role and responsibility of each care team member
- Determine which types of patients NPs and PAs can manage independently and/or in collaboration with a physician
- Codify clinical and nonclinical expectations for NPs, PAs, and physicians
- Identify changes within current support staff roles and expectations necessary to support alternative care delivery models (clinical RN, clinical care coordinator [CCC], MA, scribe, navigator, and so on)
- Identify changes in the current infrastructure necessary to support enhanced care models (for example, scheduling templates, onboarding programs, leadership roles, electronic health records, and so on)
- Develop an implementation plan to drive care model changes.

As the final step in the redesign phase, physicians and NP and PA leaders together presented workgroup recommendations to the steering committee and outlined the support each department needed to help ensure success. Each team updated the steering committee monthly on progress and outcomes of the implementation plan.

The institutional review board determined that this project did not involve human subjects, and the study was therefore exempt.

## RESULTS

All three specialties identified meaningful workflow changes to improve patient access (ambulatory practice) and coverage/throughput (inpatient). Table 1 highlights the change process activities and Table 2 outlines the SMART goals and metrics identified by the teams.

**TABLE 1.** Specialty implementation plans

**Outpatient cardiology**
□ Standardize work effort and expected patient-facing hours/administrative time. □ Standardize scheduling templates. □ Create uniformity related to staffing patients with APPs. □ Provide education related to billing of non-face-to-face encounters. □ Create standardization and processes across support roles. □ Provide documentation and coding training. □ Create monthly face-to-face meetings with clinical APPs and administration to review productivity. □ Review workflows of prescription refills for physicians, APPs, and RNs. □ Align APP incentives to include patient-facing hours and/or volume.
**Inpatient cardiology**
□ Identify patient populations most appropriate for APP rounding or co-management and pilot APP model wherein patients are rounded on by APPs independently. □ Create a process for APP-led service and codify the staffing model with cardiologists. □ Create protocols for APP low-risk chest pain observation service. □ Review staffing needs for coverage of APP-led service. □ Investigate alternative options for the APP “quarterback” role. □ Create monthly face-to-face meetings with clinical APPs and administration to review productivity.
**Hospital medicine**
□ Transition one ED admitter role to a cross-cover role. □ Provide clarity for RNs on urgent versus routine calls at night. □ Develop a process for improved utilization of routine/urgent notation in PerfectServe. □ Provide clarification related to the role of APPs on cross-cover and process used by RNs to contact service. □ Improve documentation for critical care and prolonged visits related to cross-cover notes. □ Perform annual review of all APPs inclusive of a professional development plan. □ Identify opportunities for APPs to support incremental coverage activities (for example, rounding, diabetic consults, substance abuse service, orthopedics co-management, long stay service, transitional care support, and so on).
**Oncology**
□ Standardize work effort and expected patient-facing hours/administrative time. □ Standardize scheduling templates. □ Streamline laboratory collection before chemotherapy at the University Campus. □ Develop consistent guidelines for visits seen independently across the service line. □ Review open chemotherapy chair times and scheduling process. □ Create monthly face-to-face meetings with APPs and administration to review productivity.

**TABLE 2.** Proposed SMART goals and metrics

Outpatient cardiology	Inpatient cardiology	Hospital medicine	Oncology
New patient visits (third next available)	Time to consult to signed	Time from ED consult to bed of <90 min in ≥85% of ED admissions	New patient appointment by location (third next available)
Total OP volume	Acute myocardial infarction mortality	APP wRVUs	Days from referral to new appointment for oncology
New patient volume	Increase monthly daytime patient volume to 100 patients per month per APP	APP new encounter volume per month per full-time employee	Days from referral to new appointment for hematology
New patient no-show rate	Increase monthly night shift volume to 50 patients per month per APP	APP turnover (including full-time moving to PRN)	APP total billable visit volume per month
New patient slots not filled	Time to consult to signed	APP cross-cover encounters per month	Oncologist total billable visit volume per month
No-show rate for hospital follow-up		Unique PerfectServe messages volume per month	New patient appointment by location (third next available)
% of APPs to have 30 patient-facing hours available per week		Average admissions per APP shift	Days from referral to new appointment for oncology
APP encounters per month			

Examples of key recommended changes include:

- **Outpatient cardiology:** Developed standardized scheduling templates to match goals for organizational patient-facing hour expectations; created daily rapid access appointments, thereby improving access; and allowed for daily administrative time. Additionally, the group identified the need for increased support staff. Reducing the ratio of NPs and PAs to MAs improved NP and PA productivity and job satisfaction by allowing individuals in those roles to delegate appropriate tasks to MAs. Additional documentation and coding training was offered with posttraining efficiency improvement documented. Finally, clarification of staff duties, such as prescription refill protocols, allowed NPs and PAs to see additional patients requiring management for chronic conditions, posthospital follow-up visits within 7 days, and appointments for new complaints. This also increased patient satisfaction and minimized avoidable ED visits.
- **Inpatient cardiology:** Identified patient populations most appropriate for NP and PA rounding or co-management and piloted a new model wherein patients are rounded on by NPs and PAs independently. Additionally, the group plans to develop a low-risk chest pain observation service led by NPs and PAs.
- **Hospital medicine:** With a high cross-cover census (more than 200 patients per night), a plan was developed to transition one ED admitter role to a cross-cover role. Additional training will be provided for RNs on distinguishing between urgent and routine calls at night as well as on using the appropriate notation (routine versus urgent) in the notification system. The group identified opportunities for NPs and PAs to support incremental coverage activities, such as rounding, diabetic consults, orthopedic co-management, and transitional care support.
- **Oncology:** Developed standardized scheduling templates, patient-facing hours expectations, and administrative time. Additionally, the group identified the need for training on documentation and coding.

**FIGURE 2.**
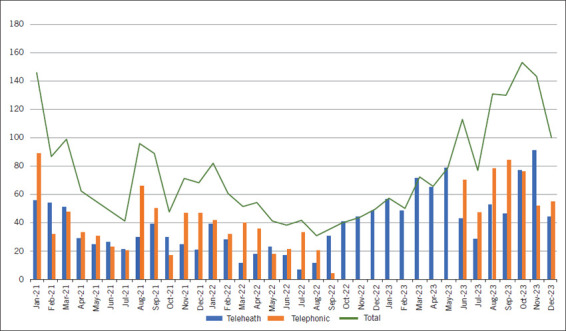
Cardiology telehealth encounters trend

**FIGURE 3.**
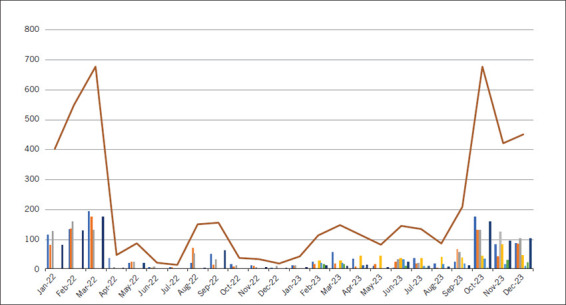
Inpatient APP cardiology encounters by provider. Colored bars represent the number of encounters per individual provider; brown line represents total encounters.

**FIGURE 4.**
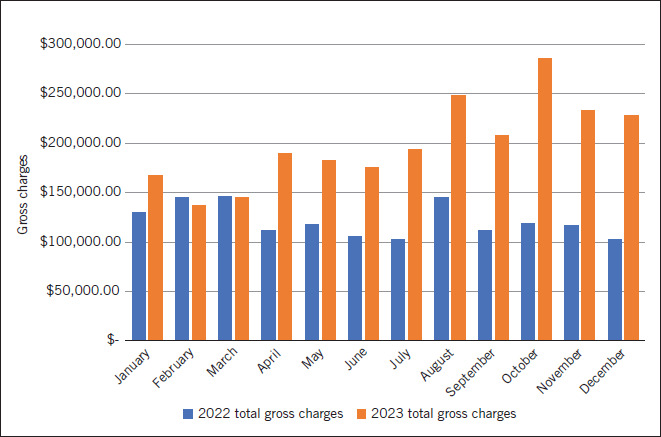
Hospital medicine APP gross charges: 2022 versus 2023

Using the implementation plans, the organization developed a project team to help support and track progress within the individual specialty teams. The organization was able to:

- Improve access to subsequent and urgent outpatient visits for patients
  - NP and PA in-person encounters in outpatient cardiology increased by 7.5% in the first 6 months.
  - NP and PA telehealth visits in outpatient cardiology increased by 93% within the first 6 months (Figure 2).
- Increase the inpatient cardiology footprint by developing an NP- and PA-led service, whereby encounters increased from fewer than 100 to between 500 and 700 (Figure 3). This improved NP and PA retention and satisfaction.
- Improve the NP and PA overnight cross-coverage census by 50%.
- Reduce overall NP and PA turnover in hospital medicine.
- Increase documented NP and PA critical care visits by more than 300 in 6 months, which resulted in more accurate reporting and a doubling of NP and PA gross charges per month in hospital medicine (Figure 4).

Oncology had positive conversations, but the group was unable to fully implement the recommendations or provide data based on changes in physician and administrative leadership in the specialty. The specialty was beginning to implement these changes at the time this article went to press.

In addition, the organization has demonstrated greater NP and PA satisfaction and engagement, including a reduction in turnover by 13%, within the specialties that underwent redesign. Other specialties have approached leadership to discuss participating in this process.

An evaluation of the process and project identified several opportunities or recommendations that could be pursued in future iterations:

- Provide data upfront to better demonstrate the need for change.
- Improve clarity related to project goals and project expectations.
- Add protected time for specialty stakeholders to perform implementation work.
- Secure project team resources or personnel such as data analysts, nonprovider project managers, and administrative support or scheduling help.

## DISCUSSION

This organizational case report builds on one previously published in the *Physician Leadership Journal*, which demonstrated a 17% increase in NP and PA encounters after further analysis and an intentional care model redesign focused on NP and PA utilization.^3^ Using a similar approach, a three-hospital system increased its monthly encounters by more than 6,600 across nine specialties.^5^

NPs and PAs can provide a clear view of the care team, as they often bridge the intersection between provider and staff-based roles. The optimization of NP and PA roles often leads to the optimization of other care team roles, including those of the physician, RN, MA, and care coordinator. An objective analysis followed by specialty-specific discussions can have a meaningful impact on patient access to care through NPs and PAs and can help organizations enhance performance and drive revenue without increasing overall costs. Other studies have shown comparable results using different frameworks; however, all have reported use of an intentional approach focused on the optimization of the NP and PA.^3^,^6^,^10^,^11^

Although much has been made of NP or PA scope of practice expansion at the state level, data are mixed on whether these changes impact overall NP or PA productivity.^7^ For example, in 2022, Stefos and colleagues identified differences in productivity by profession (NP and PA) and region despite having similar regulations.^12^ Often, organizational bylaws and culture, historical practice, and compensation can have a much larger impact on NP and PA utilization.^8^,^9^ Using an intentional process with the support of executives, physicians, and NP and PA leaders can help organizations implement specific practices, policies, and procedures designed to enhance the performance of the entire care team. It is crucial to recognize the value of both NPs and PAs and their critical role in transforming care delivery, increasing access, and delivering positive financial results.

In care model optimization, several factors can promote success. These measures include developing clearly defined expectations of all team members; ensuring tools and training are in place to support the team during role changes; involvement of engaged and accountable specialty and executive leadership; delivering data on performance targets to the team regularly; aligning communication strategy; understanding the total cost of delivering care; and aligning compensation programs between physicians and APPs.

### Limitations

Several limitations exist related to implementation. First, though improvements in both NP and PA encounters were noted, it is still being determined if the differences will be lasting or statistically significant. Additionally, although the solutions were designed for NPs and PAs collectively, it is uncertain if the changes in responsibilities or roles had a disproportionate effect on either NPs or PAs specifically based on study design. The organization described in this case report has bylaws that are equivalent for NPs and PAs and is located in a state in which NPs and PAs have similar scopes of practice by law. Differences in the NP and PA roles in this context could be a potential area for further research in care team optimization, particularly in states in which NPs and PAs have differing scopes and in organizations in which those differing scopes are reflected.

In this study, the organization's senior leadership identified specific specialties within which to focus on care team optimization, as these specialties were perceived as having a higher readiness to change than others and were also important in the context of the organization's overall strategy to enhance patient care access and promote alignment with organizational goals. During the analysis phase, data showed opportunities to optimize the NP and PA role in other specialties, but these specialties were not selected due to the perceived lack of readiness for change within them or their lack of alignment with overall organizational strategy. Selection bias may therefore have affected results, and as such, results may not be reproducible through the same process for all specialties. Similar processes have been used by other organizations to select specialties for this sort of study.^3^,^7^,^13^ Future research on this topic could focus on how the characteristics of specific teams impact their success in care team optimization.

These findings, reported 6 months after project completion, demonstrate some early and moderate success; however, as processes change and focuses shift, maintaining the results may be challenging. It is common to succeed in design but encounter difficulties in implementation. In addition to implementation challenges, the team encountered gaps in the literature regarding benchmarks on acceptable staffing for overnight management of patients as well as on the utilization and limitations of messaging platforms used to communicate patient needs. These gaps could lead to future research.

## CONCLUSION

Optimizing the care delivery team can help to improve patient access and the revenue contribution margin of NPs and PAs. It can also support greater provider engagement and reduce NP and PA turnover. Although organizational barriers and implementation challenges must be considered with any care model redesign, adoption of an intentional and data-driven approach increases the likelihood for success.
